# 

**Published:** 1873-12

**Authors:** 


### OHIO COLLEGE
—OF—
### Dental Surgery.

The Twenty-seventh Annual Session of the Ohio College of Dental Surgery, will commence on Tuesday, the 16th of October next, and continue until March 1st, 1873.

### FACULTY:

JAMES TAYLOR, M. D., D. D. S.,
Emeritus Professor of Institutes of Dental Science.
C. KEARNS, M. D.,
Professor of Anatomy.
J. S. CASSIDY, D. D. S.,
Professor of Chemistry and Therapeutics.
EDWARD RIVES, M. D.,
Professor of Physiology and Histology.
F. BRUNNING, M. D.,
Professor of Pathology and Therapeutics.
J. TAFT, D. D. S.,
Professor of Operative Dentistry and Dental Hygiene.
C. PALMER, D. D. S.,
Professor of Clinical and Mechanical Dentistry.
WILL TAFT, D. D. S,
Professor of Clinical Dentistry.
A. SCHWAGMEYER, M. D.,
Demonstrator of Anatomy.

### TEXT BOOKS

Gray's and Wilson's Anatomy; Dalton's or Carpenter's Physiology; Williams' Pathology; Fown's, Turner's, Graham's, or Brandt and Taylor's Chemistry; Taft's Operative Dentistry; Richardson's Mechanical Dentistry; Harris' Principles and Practce of Dental Science, Tomes Dental Surgery, Watts' Chemical Essays, Harris' Dental Dictionary.

### TERMS OF ADMISSION

Tickets must be procured at the beginning of the session.

| | |
|---|---|
| Tickets for the course, - - - - | $100 00 |
| Demonstrator's Ticket for Anatomy, - - | 5 00 |
| Matriculation Fee, but once, - - - | 5 00 |
| Diploma Fee, - - - - - - | 30 00 |

### TERMS OF GRADUATION:

Two courses, (including one of the dissections,) with a satisfactory dental pupilage.

Eight years reputable practice, or a satisfactory examination upon the branches of the Junior Course in this Institution will be received as equal to one course.

An acceptible thesis upon some subject on Dental science.

Must possess a good moral character, and be twenty-one years old.

For further information address, J. TAFT, *Dean.*
117 West Fourth Street, Cincinnati

### NEW ORLEANS DENTAL COLLEGE,

**158 Canal Street, Corner of Baronne.**

**1872-73**............SIXTH ANNUAL SESSION............**1872-73**

### FACULTY.

**WM. S. CHANDLER, D. D. S.,** Emeritus Professor of Operative Dental Surgery

---

**JAS. S. KNAPP, D. D. S.,** Professor of Theory and Practice.

**ANDREW F. McLAIN, M. D., D. D. S.,** Professor of Institutes of Medicine and Dentistry and Special Therapeutics.

**CHAS. E. KELLS, D. D. S.,** Professor of the Science of Dental Mechanism.

**S. P. CUTLER, M. D., D. D. S.,** Professor of Chemistry, Mineralogy, Microscopy and Histology.

**JOHN G. ANGELL, D. D. S.,** Professor of Operative Dental Surgery.

**JOHN A. ARRINGTON, D. D. S.,** Professor of Clinical Dentistry and Dental Materia Medica.

**ALFRED W. PERRY, M. D.,** Professor of Anatomy and Physiology.

The **Sixth Regular Course of Lectures** of the NEW ORLEANS DENTAL COLLEGE, for the Session of 1872–73, will commence on the eighteenth of November, and continue four months.

The superior advantages of this location for a Dental College should not be forgotten by Students who live in a colder climate, and who, by coming here, will avoid the severity and rigors of a Northern winter, and enjoy four months of comparatively mild and pleasant weather. To those in delicate health this is an inducement of no inconsiderable importance.

☞ The Faculty is composed entirely, with one exception, of gentlemen in active Dental practice.

| | |
|---|---|
| Professors' Tickets, for a full course | **$100** |
| Matriculation Fee, (paid once) | **5** |
| Diploma Fee | **30** |

Board and lodging can be procured as cheaply in New Orleans as any large city in the country. For further information, address the Dean of the Faculty,

**JAS. S. KNAPP, D. D. S.,**
Office, No. **15** Baronne street, New Orleans.

**Or the Secretary,**
Prof. A. F. McLAIN, M. D., D. D. S.,
Acting Dean, Box **834**.

AMERICAN DENTAL ASSOCIATION—Meets on the first Tuesday in August 1873, at Put-in-Bay. *Pres.* P. C. G. Hunt, Indianapolis, Ind. *Rec. Sec.* M. S. DEAN, Chicago.

AMERICAN DENTAL CONVENTION.—Meets at Saratoga Springs; second Tuesday in Aug. 1872. *Pres.* Dr. I. J. Wetherbee, Boston. *Sec.* C. S, Hurlburt, Springfield, Mass.

*List of Societies represented in the American Dental Association.*

AMERICAN ACADEMY OF DENTAL SCIENCE—Meets at Boston, Mass. *Pres.* DANIEL HARWOOD. M. D., of Boston. *Sec.* L. D SHEPARD, D. D. S., of Boston. Cor. Sec. E. N. Harris

BROOKLYN DENTAL SOCIETY—Meets semi-monthly. *Pres.* Wm Jarvie, jr. *Rec Sec.* C. P. Crandell. *Cor. Sec.* C. A. Marvin.

BUFFALO DENTAL SOCIETY — Meets second Monday of each month at Buffalo. *Pres.* S. A. FREEMAN. *Rec. Sec.* G C. Daboll,

BOSTON DENTAL INSTITUTE. Meets first Tuesday of each month. *Pres.* D. G. Williams. *Cor. Sec.* D S. Dickerman

CALIFORNIA STATE DENTAL ASSOCIATION.—Meets annually in June. *Pres.* Wm. Dutch. San Francisco. *Rec. Sec.* H. J PLOMTEAUX, Woodland.

CINCINNATI DENTAL SOCIETY—Meets semi-monthly at Cincinnati. *Pres.* Geo. WATT, *Rec. Sec.* Wm. TAFT.

CHICAGO DENTAL SOCIETY—Meets monthly at Chicago. *Pres.* I. N. Cranse *Rec Sec.* EDGAR D. SWAIN

CONNECTICUT VALLEY DENTAL ASSOCIATION—Meets semi-annually second Tuesday of May and October. *Pres.* A. A. HOWLAND, Barre, Vt. *Rec. Sec.* W. H. JONES, o Boston, Mass.

CONNECTICUT STATE DENTAL ASSOCIATION—*Pres.* H. L. SAGE, Bridgeport *Cor. Sec.* JOHN CODY, Hartford. *Rec. Sec* C. A POWERS, Hartford.

CHARLESTON DENTAL ASSOCIATION.—Meets second Tuesday of every month *Prest.* T. F. CHUPEIN. *Sec. and Treas* B. A. MUCKENFUSS.

DELAWARE DENTAL ASSOCIATION—Meets semi-annually. *Pres* E. W. HAINES, Newark. *Sec.* C. R. JEFFRIES. Wilmington.

EASTERN OHIO DENTAL ASSOCIATION—Meets on Tuesday, Februery 27th, 1872, at Alliance. *Pres.* J. C. WHINERY *Sec.* D. B. MCLAIN.

EAST TENNESSEE DENTAL ASSOCIATION—Meets annually at Knoxville. Tenn *Pres* WM. H. COOKE, of Cleveland. *Rec. Sec.* A. P. WHITE, Knoxville.

FOREST CITY SOCIETY OF DENTAL SURGEONS. *Pres.* B. Strickland, Cleveland. *Rec. Sec.*, H. L Ambler. Cleveland. *Cor. Sec.* Chas. Buffett, Cleveland.

GEORGIA STATE DENTAL SOCIETY—Next meeting at Atlanta, July 30th, 1870. *Pres.* F. Y. CLARK; *Cor. Sec.* J. P. H. BROWN, Augusta.

HARRIS DEMTAL ASSOCIATION—Meets quarterly on the first Thursday in February, May, August and November. Pres. J. A. Martin, Strasburg, Sec. Wm. N. Amer, Lancaster

HUDSON RIVER ASSOCIATION OF DENTAL SURGEONS—Meets on the second Wednesdays of May, September and January. *Pres.* L. S. STRAW, Newburg N.Y. *Rec. Sec* T. W. DUBOIS. Pougkeepsie, N. Y.

HUDSON VALLEY DENTAL ASSOCIATION—meets monthly at Troy, N Y. *Pres.* L. C WHEELER, *Rec. Sec.* S. G. ANDRES. *Cor. Sec* S. D. FRENCH.

ILLINOIS STATE DENTAL SOCIETY—Meets annually. *Pres.* J. N. Crouse, Chicago, Sec. C. R. E. Coch, Chicago. Meets second Tuesday in May, 1873 at Rock Island.

INDIANA STATE DENTAL SOCIETY—Meets next year at New Albany on the first Tuesday of June. *Pres* J Richardson, Terre Haute. *Cor. and Rec. Sec* P. McDonald, Goshen.

IOWA STATE DENTAL SOCIETY—Meets annually. *Next meeting at Davenport, on the second Tuesday of May. *Pres.* L. C. Ingersoll, Keokuk. *Rec. Sec.* P. E. White, of Davenport. *Cor Sec.* A. J. Redrick, Sioux City.

KANSAS STATE DENTAL SOCIETY—Meets semi-annually. Next meeting at Topeka, Sept. 12, 1871 the next at Atchison, May 2. 1872. *Pres.* J. B. WHEELER. *Sec.* E. C. Fuller.

KANSAS & MISSOURI VALLEY DENTAL ASSOCIATION—Meets semi-monthly. Pres. J. R. Stark. Sec. J. E. Cravens.

KENTUCKY STATE DENTAL ASSOCIATION—Meets annually, and Semi monthly, in the City of Louisville. Annual meeting. First Tuesday in June, at 2 o'clock P. M. Semi-monthly. meetings 2d and 4th Tuesday Nights. W. H. Goddard, *Pres't.* G. W. Priest, *Sec*, Both at Louisville,

LAKE ERIE DENTAL ASSOCIATION.—Meets Annually.* *Pres.* J. G. TEMPLETON. *Sec.* C. B. ANSART. Next meeting first Tuesday of May, 1872, at Conneautville, Pa.

MAD RIVER VALLEY DENTAL SOCIETY—Meets at Dayton on the first Tuesdays of April and October. Pres. G. Watt, Xenia, Sec. D. G. French, Xenia.

MAINE DENTAL SOCIETY—Meets in August, prox., at Augusta. *Pres.* H. Leavitt Skowhegan. *Sec.* C. E. Hussey, Biddleford.

MARYLAND ASSOCIATION OF DENTISTS—Meetsthe last Thursday of every month. *Pres.* J. B. BEAN, *Sec.*, S M. FIELD.

MASSACHUSETTS DENTAL SOCIETY—Meets monthly. *Pres.* J. H. Batchelder, of Salem. *Sec.* C. Wilson, *Cor. See.* L. D. Shepard

MICHIGAN STATE DENTAL ASOCIATION—Meets annually 2d Tuesday of Oct. at Detroit. *Pres.* R. G. Thomasr Detroit. *Rec. and Cor. Sec.* T. R] Perry of Grand Rapids.

MICHIGAN CENTRAL DENTAL ASSOCIATION—Meets annually at Jackson, second Wednesday of January, 1873. Pres, C. C. Jenkins, Ann Arbor. *Sec.* W. H. Darrance, Jackson.

MISSISSIPPI VALLEY DENTAL ASSOCIATION—Meets annually at Cincinnati, on first Wednesday in March *Pres* F. H. REHWINKLE Chillicothe *Rec. Sec.* F HUNT Cincinnati, O.

MINNESOTA DENTAL ASSOCIATION—Meets at Minneapolis last Wednesday in Jun 1873. Pres. D. C. Price, St. Paul. Sec. C. D. Montreville, St. Paul.

MISSOURI DENTAL ASSOCIATION—Meets on the first Tesday of June, in St Joseph *Pres.* J. K. Stark, Kansas City, *Sec.* W. H. EAMES, St. Louis.

MISSOURI VALLEY DENTAL SOCIETY.* Meets on the second Tuesday in July and January. *Pres.* E. J. WOODBURY of Council Bluffs, Iowa *Sec. and Treas.* J. F. Sanborn Tabor, Iowa.

MERRIMAC VALLEY DENTAL SOCIETY—Meets semi annually first Tuesday of Oct. and May.* *Pres.* I. H. KIDDER, Mass. *Rec. Sec.* A. M. DUDLEY, of Lowell *Cor. Sec.* D. T. PORTER, Lawrence, Mass.

MEMPHIS DENTAL SOCIETY.—Meets on the first Tuesday of each month. *Pres* W. T Arrington, *Sec.* V. F. Elliott, *Treas.* C. L. Harris.

NASHVILLE DENTAL ASSOCIATION—Meets on the second Tuesday of each month *Pres.* J C. Ross, *Sec* R R. FREEMAN. *Cor. Sec.* S. J. COBB

NEW JERSEY STATE DENTAL SOCIETY.—Meets annually. Next meeting at Long Branch, on the second Tuesday in July, 1871. *Pres.* A. M. KINGSLEY of Elizabeth, *Sec.* L M. De Lange, Bordentown.

NEW YORK STATE DENTAL SOCIETY—Meets at Albany on the last Wednesday of June next *Pres.* C. A Marvin of Brooklyn. *Sec.* CHAS. BARNS, of Syracuse. *Cor. Sec.* S. A. Freeman. *State Censors*, S. H. MCCALL, Binghamton, 6th Dist. R. G SNOW, Bu falo, 8th Dist.

NEW HAVEN DENTAL SOCIETY—Meets on the first Wednesday of each month New Haven. *Pres.* J. T. METCALF. *Rec. and Cor. Sec.* C. L. SMITH, of New Haven.

NORTHERN OHIO DENTAL ASSOCIATION—Meets annually, (Second Tuesday o June 1873) at Put in Bay. *Pres* A. TERRY, Norwalk; *Rec. Sec.* A. F. PRICE, Fremont.

NORTHERN IOWA DENTAL ASSOCIATION—Meets annually, on the 2d Tuesday in June, 1870. Next meeting at Waterloo. *Pres.* J. T. ABBOTT, Manchester. *Rec. Sec* A. V. EATON, Anamosa. *Cor. Sec.* A B. MASON, Waterloo.

OHIO DENTAL COLLEGE ASSOCIATION—Meets annually in March, at Cincinnat *Pres.* W H. GODDARD. Louisville, Ky. *Rec Sec.* J TAFT, of Cincinnati.

OHIO STATE DENTAL ASSOCIATION.—Meets annually, at Columbus. [Next meeting—first Wednesday of December.] *Pres.* L. Bippert, Cleveland. *Rec. Sec.* C. R. TAFT. Mansfield, O. *Cor. Sec.* C. R BUTLER, Cleveland, O.

ODONTOGRAPHIC SOCIETY OF PENNSYLVANIA—Meets monthly in Philadelphia Next meeting, Wednesday, Sept 1st *Pres.* T. C. Stellwagen. *Rec. Sec.* A. BOICE *Cor. Sec.* W. F. Litch.

ODONTOLOGICAL SOCIETY OF NEW YORK CITY—Meets the second Tuesday of each month.* *Pres.* C E. Francis *Recs. Sec.* W. E. Hoag.

OLD COLONY DENTAL ASSOCIATION — Meets quarterly.* *Pres.* C. G. Davis *Req. Sec.* L. W. PUFFER, North Bridgewater. Mass. *Cor. Sec.* N. C. FOWLER, Yarmouth, Mass.

PENNSYLVANIA STATE DENTAL SOCIETY—Meets next in Lancaster. second Tuesday of July, 1873. *Pres.* M. E Gillespie, Pittsburgh. *Rec. Sec* C. B. Ansart, Oil City. *Cor. Sec.* S. WELCHENS, Lancaster.

SAN FRANCISCO DENTAL ASSOCIATION.—Meets monthly. *Pres.* HENRY AUSTIN *Cor. Sec.* H. E. KNOX *Rec. Sec.* A. B. WOOD.

POUGHKEEPSIE DENTAL SOCIETY.—Meets monthly, *Pres.* J. H. Mann, *Sec.* H. F Clark.

ST. LOUIS DENTAL SOCIETY—Meets monthly. *Pres.* W. H. Morrison, *Sec.* A. H. Fuller

SUSQUEHANNA DENTAL ASSOCIATION—Meets annually*. *Pres.* H. H. Morton, *Rec. Sec.* G. W. Klump, Williamsport. Meets Nov. 12. 1873.

SOCIETY OF DENTAL SURGEONS OF THE CITY OF NEW YORK—Meets semi monthly in New York. *Pres.* B. W FRANKLIN. *Rec. Sec.* J. S LATIMER. *Cor. Sec* T. H. BURRAS.

SOUTHERN DENTAL ASSOCIATION.—Meets annually.* Next meeting the last Tuesday of July, 1872, at Richmond, Va. *Pres.* F. Y. Clark, Savannah, S. C. *Rec. Sec* O. J. Bond, New Orleans.

SOUTH CAROLINA STATE DENTAL ASSOCIATION.—Meets semi annually in Ma and Nov. *Pres.* W. C. WARDLAW Abbeville; *Sec* O. J. BOND, Marion

PENNSYLVANIA ASSOCIATION OF DENTAL SURGEONS—Meets annually, in Philadelphia *Pres.* ———, *Sec.* ———.

TENNESSEE DENTAL ASSOCIATION.—Meets semi-annually. Next meeting in Nashville, Oct. 3, 1872. *Pres.* J. C. Ross, Nashville. Sec. L. G. Noel. Nashville.

VIRGINIA STATE DENTAL ASSOCIATION—Meets in Richmond, 1st Thursday in Nov. of each year. Pres. H. M. Grant, Abington. Sec. G. F. Keesee. Richmond.

WASHINGTON CITY DENTAL SOCIETY—Meets monthly. *Pres.* R. F. HUNT. *Sec.* Dr. EVANS.

WABASH VALLEY DENTAL ASSOCIATION.—Meets annually. Next meeting at Wayne, third Tuesday in March, 1872. *Pres.* A. B. CUNNINGHAM, Attica; *Sec.* S B. BROWN, Fort Wayne.

WISCONSIN STATE DENTAL ASSOCIATION—Meets semi-annually.* Next meeti at Watertown, second Tuesday of January, 1872. *Pres* EDGAR PALMER, La Cro *Sec.* C C. CHITTENDEN, Madison.

**Place of meeting fixed by appointment.*

### DISTRICT DENTAL SOCIETIES OF THE STATE OF NEW YORK.

*1ST DISTRICT DENTAL SOCIETY.— Meets monthly in New York. Annual meeting second Wednesday in June. *Pres.* E. A. Bogne, New York. *Sec.* J S Latimer. N. Y.

2D DISTRICT DENTAL SOCIETY.—Meets quarterly in Brooklyn. Annual meeting 2d Tuesday in April. *Pres.* O. E. Hill, Brooklyn; *Rec. Sec.* N. E. Elmandorf, Brooklyn. *Cor. Sec.* L. S. Straw, Newburg

3D DISTRICT DENTAL SOCIETY.—Meets at Albany semi-annually and annually. *Pre* D. D. Frendh, Troy. *Sec.* S. J. Andress, of Albany.

*4TH DISTRICT DENTAL SOCIETY.—Meets annually in June at Saratoga. *Pres.* C. A Elmore, Saratoga Springs. *Sec.* E. S. Pearsall, Fort Edwards.

*5TH DISTRICT DENTAL SOCIETY.—Meets annually at Saratoga. *Pres.* W. W. Perkins. *Sec.* F. D. Neelans.

*6TH DISTRICT DENTAL SOCIETY.—Annual meeting at Binghampton, second Tuesday in June. *Pres.* R. Walker. *Rec. Sec.* P. L. Foote.

*7TH DISTRICT DENTAL SOCIETY.—Meets semi-annually at Rochester. *Pres.* A. G. Coleman, Canandagua. *Sec.* J. E. Line.

TH DISTRICT D NTAL SOCIETY.—Meets annually on the 2d Tuesday in May, at Buffalo. *Pres.* J. C. Gifford. *Sec.* S. A. Freeman, Buffalo.

THE 7TH AND 8TH DISTRICT SOCIETIES—Hold a union meeting on the first Tuesday of October, alternately at Buffalo and Rochester.

* Place and time of meeting fixed by appointment.

The semi-annual meeting of the Ohio State Board of Dental Examiners will be held in Columbus, Ohio, on Tuesday, Dec. 3, at ten o'clock A. M.

J. TAFT, President.

W. P. HORTON, Secretary.

### EXCHANGES.

ST. LOUIS MEDICAL AND SURGICAL JOURNAL,
BOSTON MEDICAL AND SURGICAL JOURNAL,
THE PACIFIC MEDICAL AND SURGICAL JOURNAL, SAN FRANCISCO.
BUFFALO MEDICAL AND SURGICAL JOURNAL,
PHILADELPHIA MEDICAL AND SURGICAL REPORTER,
CINCINNATI LANCET AND OBSERVER,
DENTAL COSMOS, PHILADELPHIA.
BRAITHWAITE'S RETROSPECT, N. Y.
THE DENTAL TIMES, PHILADELPHIA,
LADIES' REPOSITORY, CINCINNATI.
HALL'S JOURNAL OF HEALTH, N. Y.
CHICAGO MEDICAL EXAMINER.
THE DETROIT REVIEW OF MEDICINE AND PHARMACY.
MEDICAL RECORD, N Y.
NASHVILLE JOURNAL OF MEDICINE AND SURGERY,
AMERICAN ECLECTIC MEDICAL REVIEW, N. Y.
AMERICAN JOURNAL OF DENTAL SCIENCE, BALTIMORE.
THE UNITED STATES MEDICAL AND SURGICAL JOURNAL, CHICAGO
NEW ORLEANS JOURNAL OF MEDICINE.
WESTERN JOURNAL OF MEDICINE. INDIANAPOLIS, IND.
AMERICAN AGRICULTURIST, N. Y.
DENTAL OFFICE AND LABORATORY, PHILA.
BOSTON JOURNAL OF CHEMISTRY.
CINCINNATI MEDICAL REPERTORY.
JOURNAL OF APPLIED CHEMISTRY. N. Y.
DRUGGISTS' CIRCULAR AND CHEMICAL GAZETTE, NEW YORK.
CANADA JOURNAL OF DENTAL SCIENCE, MONTREAL.
OHIO CONVENTION REPORTER, COLUMBUS.
INDIANA JOURNAL OF MEDICINE.
JOURNAL OF THE GYNÆCOLOGICAL SOCIETY, BOSTON.
MEDICAL AND SURGICAL REPORTER. SALEM, OREGON
WOOD'S HOUSEHOLD MAGAZINE, NEWBURG, N. Y.
MISSOURI DENTAL JOURNAL, ST. LOUIS.
ECLECTIC MEDICAL JOURNAL, CINCINNATI.
INDUSTRIAL PRESS, CINCINNATI.

### OHIO COLLEGE

—OF—

### Dental Surgery.

The Twenty-seventh Annual Session of the Ohio College of Dental Surgery, will commence on Tuesday, the 16th of October next, and continue until March 1st, 1873.

### FACULTY:

JAMES TAYLOR, M. D., D. D. S.,
Emeritus Professor of Institutes of Dental Science.

C. KEARNS, M. D.,
Professor of Anatomy.

J. S. CASSIDY, D. D. S.,
Professor of Chemistry and Therapeutics.

EDWARD RIVES, M. D.,
Professor of Physiology and Histology.

F. BRUNNING, M. D.,
Professor of Pathology and Therapeutics.

J. TAFT, D. D. S.,
Professor of Operative Dentistry and Dental Hygiene.

C. PALMER, D. D. S.,
Professor of Clinical and Mechanical Dentistry.

WILL TAFT, D. D. S.,
Professor of Clinical Dentistry.

A. SCHWAGMEYER, M. D.,
Demonstrator of Anatomy.

### TEXT BOOKS

Gray's and Wilson's Anatomy; Dalton's or Carpenter's Physiology; Williams' Pathology; Fown's, Turner's, Graham's, or Brandt and Taylor's Chemistry; Taft's Operative Dentistry; Richardson's Mechanical Dentistry; Harris' Principles and Practce of Dental Science, Tomes Dental Surgery, Watts' Chemical Essays, Harris' Dental Dictionary.

### TERMS OF ADMISSION

Tickets must be procured at the beginning of the session.

| | |
|---|---|
| Tickets for the course, - - - - | $100 00 |
| Demonstrator's Ticket for Anatomy, - - | 5 00 |
| Matriculation Fee, but once, - - - | 5 00 |
| Diploma Fee, - - - - - - | 30 00 |

### TERMS OF GRADUATION:

Two courses, (including one of the dissections,) with a satisfactory dental pupilage.

Eight years reputable practice, or a satisfactory examination upon the branches of the Junior Course in this Institution will be received as equal to one course.

An acceptible thesis upon some subject on Dental science.

Must possess a good moral character, and be twenty-one years old.

For further information address, J. TAFT, *Dean*.
117 West Fourth Street, Cincinnati

### OHIO COLLEGE

—OF—

### Dental Surgery.

The Twenty-seventh Annual Session of the Ohio College of Dental Surgery, will commence on Tuesday, the 16th of October next, and continue until March 1st, 1873.

### FACULTY:

JAMES TAYLOR, M. D., D. D. S.,
Emeritus Professor of Institutes of Dental Science.

C. KEARNS, M. D.,
Professor of Anatomy.

J. S. CASSIDY, D. D. S.,
Professor of Chemistry and Therapeutics.

EDWARD RIVES, M. D.,
Professor of Physiology and Histology.

F. BRUNNING, M. D.,
Professor of Pathology and Therapeutics.

J. TAFT, D. D. S.,
Professor of Operative Dentistry and Dental Hygiene.

C. PALMER, D. D. S.,
Professor of Clinical and Mechanical Dentistry.

WILL TAFT, D. D. S.,
Professor of Clinical Dentistry.

A. SCHWAGMEYER, M. D.,
Demonstrator of Anatomy.

### TEXT BOOKS

Gray's and Wilson's Anatomy; Dalton's or Carpenter's Physiology; Williams' Pathology; Fown's, Turner's, Graham's, or Brandt and Taylor's Chemistry; Taft's Operative Dentistry; Richardson's Mechanical Dentistry; Harris' Principles and Practtce of Dental Science, Tomes Dental Surgery, Watts' Chemical Essays, Harris' Dental Dictionary.

### TERMS OF ADMISSION

Tickets must be procured at the beginning of the session.

| | |
|---|---|
| Tickets for the course, - - - - | $100 00 |
| Demonstrator's Ticket for Anatomy, - - | 5 00 |
| Matriculation Fee, but once, - - - | 5 00 |
| Diploma Fee, - - - - - - | 30 00 |

### TERMS OF GRADUATION:

Two courses, (including one of the dissections,) with a satisfactory dental pupilage.

Eight years reputable practice, or a satisfactory examination upon the branches of the Junior Course in this Institution will be received as equal to one course.

An acceptible thesis upon some subject on Dental science.

Must possess a good moral character, and be twenty-one years old.

For further information address, J. TAFT, *Dean.*
117 West Fourth Street, Cincinnati

### NEW ORLEANS DENTAL COLLEGE,

### 158 Canal Street, Corner of Baronne.

**1872-73**............SIXTH ANNUAL SESSION............**1872-73**

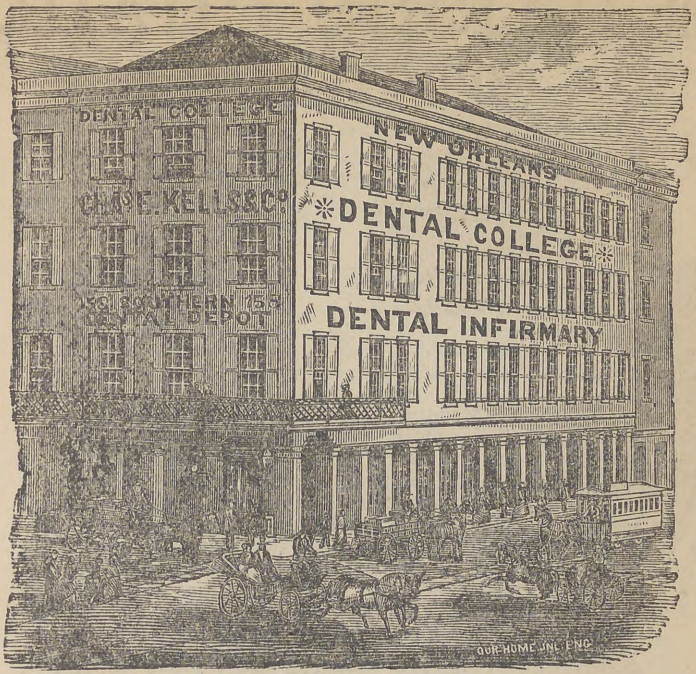

### FACULTY.

———o———

**WM. S. CHANDLER, D. D. S.,** Emeritus Professor of Operative Dental Surgery

---

**JAS. S. KNAPP, D. D. S.,** Professor of Theory and Practice.

**ANDREW F. McLAIN, M. D., D. D. S.,** Professor of Institutes of Medicine and Dentistry and Special Therapeutics.

**CHAS. E. KELLS, D. D. S.,** Professor of the Science of Dental Mechanism.

**S. P. CUTLER, M. D., D. D. S.,** Professor of Chemistry, Mineralogy, Microscopy and Histology.

**JOHN G. ANGELL, D. D. S.,** Professor of Operative Dental Surgery.

**JOHN A. ARRINGTON, D. D. S.,** Professor of Clinical Dentistry and Dental Materia Medica.

**ALFRED W. PERRY, M. D.,** Professor of Anatomy and Physiology.

The **Sixth Regular Course of Lectures** of the NEW ORLEANS DENTAL COLLEGE, for the Session of 1872–73, will commence on the eighteenth of November, and continue four months.

The superior advantages of this location for a Dental College should not be forgotten by Students who live in a colder climate, and who, by coming here, will avoid the severity and rigors of a Northern winter, and enjoy four months of comparatively mild and pleasant weather. To those in delicate health this is an inducement of no inconsiderable importance.

☞ The Faculty is composed entirely, with one exception, of gentlemen in active Dental practice.

| | |
|---|---|
| Professors' Tickets, for a full course.................................... | **$100** |
| Matriculation Fee, (paid once).......................................... | **5** |
| Diploma Fee................................................................ | **30** |

Board and lodging can be procured as cheaply in New Orleans as any large city in the country. For further information, address the Dean of the Faculty,

**JAS. S. KNAPP, D. D. S.,**
Office, No. 15 Baronne street, New Orleans.

Or the Secretary,
Prof. A. F. McLAIN, M. D., D. D. S.,
Acting Dean, Box 834.

### NEW ORLEANS DENTAL COLLEGE,

**158 Canal Street, Corner of Baronne.**

**1872-73**............SIXTH ANNUAL SESSION............**1872-73**

### FACULTY.

**WM. S. CHANDLER, D. D. S.,** Emeritus Professor of Operative Dental Surgery

**JAS. S. KNAPP, D. D. S.,** Professor of Theory and Practice.

**ANDREW F. McLAIN, M. D., D. D. S.,** Professor of Institutes of Medicine and Dentistry and Special Therapeutics.

**CHAS. E. KELLS, D. D. S.,** Professor of the Science of Dental Mechanism.

**S. P. CUTLER, M. D., D. D. S.,** Professor of Chemistry, Mineralogy, Microscopy and Histology.

**JOHN G. ANGELL, D. D. S.,** Professor of Operative Dental Surgery.

**JOHN A. ARRINGTON, D. D. S.,** Professor of Clinical Dentistry and Dental Materia Medica.

**ALFRED W. PERRY, M. D.,** Professor of Anatomy and Physiology.

The **Sixth Regular Course of Lectures** of the NEW ORLEANS DENTAL COLLEGE, for the Session of 1872–73, will commence on the eighteenth of November, and continue four months.

The superior advantages of this location for a Dental College should not be forgotten by Students who live in a colder climate, and who, by coming here, will avoid the severity and rigors of a Northern winter, and enjoy four months of comparatively mild and pleasant weather. To those in delicate health this is an inducement of no inconsiderable importance.

☞ The Faculty is composed entirely, with one exception, of gentlemen in active Dental practice.

| | |
|---|---|
| Professors' Tickets, for a full course | $100 |
| Matriculation Fee, (paid once) | 5 |
| Diploma Fee | 30 |

Board and lodging can be procured as cheaply in New Orleans as any large city in the country. For further information, address the Dean of the Faculty,

**JAS. S. KNAPP, D. D. S.,**
Office, No. **15** Baronne street, New Orleans.

Or the Secretary.

Prof. A. F. McLAIN, M. D., D. D. S.,
Acting Dean, Box **834**.

### PENNSYLVANIA COLLEGE OF DENTAL SURGERY.

THE EIGHTEENTH ANNUAL SESSION, 1873-'74.

### FACULTY.

T. L. BUCKINGHAM, D. D. S.,
*Professor of Chemistry.*
E. WILDMAN, M. D., D. D. S.,
*Professor of Mechanical Dentistry and Metallurgy.*
G. T. BAKER, D. D. S
*Professsor of Dental Pathology and Therapeutics.*
JAMES TRUMAN, D. D. S.,
*Professor of Dental Histology and Operative Chemistry.*
JAMES TYSON, M. D.,
*Professor of Physiology and Microscopic Anatomy.*
*J. EWING MEARS, M. D.,*
*Professor of Anatomy and Surgery.*
E. R. PETTIT, D. D. S.,
*Demonstrator of Operative Dentistry.*
A, B. ABELL, JR., D. D. S.,
*Demonstrator of Mechanical Dentistry.*
J. L. SAXTON, D. D. S.,
*Assistant Demonstrator of Operative Dentistry.*
A. H. HENDERSON, D. D. S.,
*Assistant Demonstrator of Mechanical Dentistry.*

The College will be open for instruction during the entire year, except the last week in August and the first week in September. In the Dispensary and Laboratory, ample opportunities will be afforded the student for the prosecution of the practical part of the profession, under the guidance and supervision of Demonstrators of known integrity and capacity. A Clinical Lecture will be given and operations performed by one of the professors every Saturday afternoon during the sessions.

### SPRING AND FALL SESSIONS.

The Spring Course of Lectures will commence on the first Monday in April, and continue uutil the last of June.

The Fall Course will commence on the second Monday in September, and continue until the last of October,

During the Spring and Fall Sessions there will be one Lecture a day, from 5 to 6 o'clock, P. M., and with the exception of Saturday, the Dispensary and Laboratory will be open daily for six hours.

The fee for the tickets will be $50.

The attendance of the entire Spring and Fall Courses will be deemed equivalent to the term of pupilage under a private preceptor during the recess of the regular sessions.

### THE REGULAR SESSION.

Will commence on the first Monday of November, and continue until the first of March ensuing. The course is so arranged that eighteen lectures will be delivered each week on the various branches taught in the College.

FEES.

| | |
|---|---|
| Matriculation, (paid but once,) | $5 00 |
| For the Course, (Demonstrator's Ticket included,) | 100 00 |
| Diploma, | 30 00 |

For further information, address,

**E. WILDMAN Dean,**

**No. 1205 Arch Street, Philadelphia.**

Board can be obtained at from $4.00 to $8.00 per week.

All the Instruments and Tools required can be procured for from $15.00 to $20.00.

### NEW ORLEANS DENTAL COLLEGE,

**158 Canal Street, Corner of Baronne.**

**1872-73**..............SIXTH ANNUAL SESSION..............**1872-73**

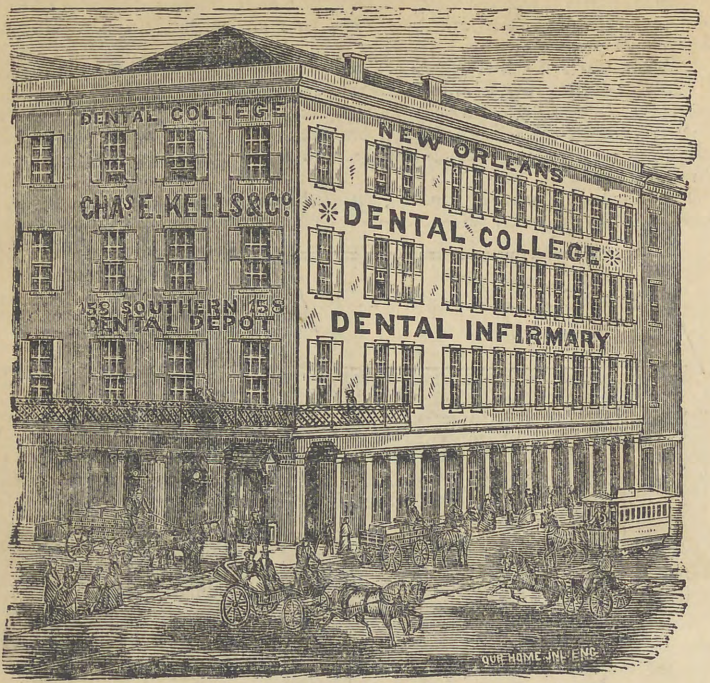

### FACULTY.

—o—

**WM. S. CHANDLER, D. D. S.,** Emeritus Professor of Operative Dental Surgery

---

**JAS. S. KNAPP, D. D. S.,** Professor of Theory and Practice.

**ANDREW F. McLAIN, M. D., D. D. S.,** Professor of Institutes of Medicine and Dentistry and Special Therapeutics.

**CHAS. E. KELLS, D. D. S.,** Professor of the Science of Dental Mechanism.

**S. P. CUTLER, M. D., D. D. S.,** Professor of Chemistry, Mineralogy, Microscopy and Histology.

**JOHN G. ANGELL, D. D. S.,** Professor of Operative Dental Surgery.

**JOHN A. ARRINGTON, D. D. S.,** Professor of Clinical Dentistry and Dental Materia Medica.

**ALFRED W. PERRY, M. D.,** Professor of Anatomy and Physiology.

The **Sixth Regular Course of Lectures** of the NEW ORLEANS DENTAL COLLEGE, for the Session of 1872-73, will commence on the eighteenth of November, and continue four months.

The superior advantages of this location for a Dental College should not be forgotten by Students who live in a colder climate, and who, by coming here, will avoid the severity and rigors of a Northern winter, and enjoy four months of comparatively mild and pleasant weather. To those in delicate health this is an inducement of no inconsiderable importance.

☞ The Faculty is composed entirely, with one exception, of gentlemen in active Dental practice.

| | |
|---|---|
| Professors' Tickets, for a full course | $100 |
| Matriculation Fee, (paid once) | 5 |
| Diploma Fee | 30 |

Board and lodging can be procured as cheaply in New Orleans as any large city in the country. For further information, address the Dean of the Faculty,

**JAS. S. KNAPP, D. D. S.,**
Office, No. 15 Baronne street, New Orleans.

Or the Secretary, Prof. A. F. McLAIN, M. D., D. D. S.,
Acting Dean, Box 834.

### PENNSYLVANIA COLLEGE OF DENTAL SURGERY.

THE EIGHTEENTH ANNUAL SESSION, 1873-'74.

### FACULTY.

T. L. BUCKINGHAM, D. D. S.,
*Professor of Chemistry.*
E. WILDMAN, M. D., D. D. S.,
*Professor of Mechanical Dentistry and Metallurgy.*
G. T. BAKER, D. D. S
*Professsor of Dental Pathology and Therapeutics.*
JAMES TRUMAN, D. D. S.,
*Professor of Dental Histology and Operative Chemistry.*
JAMES TYSON, M. D.,
*Professor of Physiology and Microscopic Anatomy*
*J. EWING MEARS, M. D.,*
*Professor of Anatomy and Surgery.*
E. R. PETTIT, D. D. S.,
*Demonstrator of Operative Dentistry.*
A, B. ABELL, JR., D. D. S.,
*Demonstrator of Mechanical Dentistry.*
J. L. SAXTON, D. D. S.,
*Assistant Demonstrator of Operative Dentistry.*
A. H. HENDERSON, D. D. S.,
*Assistant Demonstrator of Mechanical Dentistry.*

The College will be open for instruction during the entire year, except the last week in August and the first week in September. In the Dispensary and Laboratory, ample opportunities will be afforded the student for the prosecution of the practical part of the profession, under the guidance and supervision of Demonstrators of known integrity and capacity. A Clinical Lecture will be given and operations performed by one of the professors every Saturday afternoon during the sessions.

### SPRING AND FALL SESSIONS.

The Spring Course of Lectures will commence on the first Monday in April, and continue uutil the last of June.

The Fall Course will commence on the second Monday in September, and continue until the last of October,

During the Spring and Fall Sessions there will be one Lecture a day, from 5 to 6 o'clock, P. M., and with the exception of Saturday, the Dispensary and Laboratory will be open daily for six hours.

The fee for the tickets will be $50.

The attendance of the entire Spring and Fall Courses will be deemed equivalent to the term of pupilage under a private preceptor during the recess of the regular sessions.

### THE REGULAR SESSION.

Will commence on the first Monday of November, and continue until the first of March ensuing. The course is so arranged that eighteen lectures will be delivered each week on the various branches taught in the College

FEES.

| | |
|---|---|
| Matriculation, (paid but once,) | $5 00 |
| For the Course, (Demonstrator's Ticket included,) | 100 00 |
| Diploma, | 30 00 |

For further information, address,

**E. WILDMAN Dean,**

**No. 1205 Arch Street, Philadelphia.**

Board can be obtained at from $4.00 to $8.00 per week.

All the Instruments and Tools required can be procured for from $15.00 to $20.00.

### PENNSYLVANIA COLLEGE OF DENTAL SURGERY.

THE EIGHTEENTH ANNUAL SESSION, 1873-'74.

### FACULTY.

T. L. BUCKINGHAM, D. D. S.,
*Professor of Chemistry.*
E. WILDMAN, M. D., D. D. S.,
*Professor of Mechanical Dentistry and Metallurgy.*
G. T. BAKER, D. D. S
*Professsor of Dental Pathology and Therapeutics.*
JAMES TRUMAN, D. D. S.,
*Professor of Dental Histology and Operative Chemistry.*
JAMES TYSON, M. D.,
*Professor of Physiology and Microscopic Anatomy*
*J. EWING MEARS, M. D.,*
*Professor of Anatomy and Surgery.*
E. R. PETTIT, D. D. S.,
*Demonstrator of Operative Dentistry.*
A, B. ABELL, JR., D. D. S.,
*Demonstrator of Mechanical Dentistry.*
J. L. SAXTON, D. D S.,
*Assistant Demonstrator of Operative Dentistry.*
A. H. HENDERSON, D. D. S.,
*Assistant Demonstrator of Mechanical Dentistry.*

The College will be open for instruction during the entire year, except the last week in August and the first week in September. In the Dispensary and Laboratory, ample opportunities will be afforded the student for the prosecution of the practical part of the profession, under the guidance and supervision of Demonstrators of known integrity and capacity. A Clinical Lecture will be given and operations performed by one of the professors every Saturday afternoon during the sessions.

### SPRING AND FALL SESSIONS.

The Spring Course of Lectures will commence on the first Monday in April, and continue uutil the last of June.

The Fall Course will commence on the second Monday in September, and continue until the last of October,

During the Spring and Fall Sessions there will be one Lecture a day, from 5 to 6 o'clock, P. M., and with the exception of Saturday, the Dispensary and Laboratory will be open daily for six hours.

The fee for the tickets will be $50.

The attendance of the entire Spring and Fall Courses will be deemed equivalent to the term of pupilage under a private preceptor during the recess of the regular sessions.

### THE REGULAR SESSION.

Will commence on the first Monday of November, and continue until the first of March ensuing. The course is so arranged that eighteen lectures will be delivered each week on the various branches taught in the College

FEES.

| | |
|---|---|
| Matriculation, (paid but once,) | $5 00 |
| For the Course, (Demonstrator's Ticket included,) | 100 00 |
| Diploma, | 30 00 |

For further information, address,

**E. WILDMAN Dean,**

**No. 1205 Arch Street, Philadelphia.**

Board can be obtained at from $4.00 to $8.00 per week.

All the Instruments and Tools required can be procured for from $15.00 to $20.00.

### OHIO COLLEGE
OF
### DENTAL SURGERY,

The Twenty-eighth Annual Session of the Ohio College of Dental Surgery will commence on Monday, the 15th of October next, and continue until March following.

### FACULTY.

JAMES TAYLOR, M. D., D. D S,
Emeritus Professor of Institutes of Dental Science.
C. KEARNS, M. D.,
Professor of Anatomy.
J. S. CASSIDY, D. D. S.,
Professor of Chemistry and Therapeutics.
EDWARD RIVES, M. D.,
Professor of Physiology and Histology.
E. BRUNNING, M. D.,
Professor of Pathology and Therapeutics.
J. TAFT, D. D. S.,
Professor of Operative Dentistry and Dental Hygiene.
FRANK HUNTER, D. D. S.,
Professor of Mechanical Dentistry.
JAMES I. TAYLOR, Jr., D. D. S.,
Professor of Clinical Dentistry.

### TEXT BOOKS.

Gray's and Wilson's Anatomy, Dalton's or Carpenter's Physiology; William's Pathology, Fowne's, Turner's, Graham's, or Brandt and Taylor's Chemistry; Taft's Operative Dentistry; Richardson's Mechanical Dentistry; Harris' Principles and Practice of Dental Science; Tomes' Dental Surgery; Watt's Chemical Essays; Harris' Dental Dictionary.

### TERMS OF ADMISSION.

Tickets must be procured at the beginning of the session.

| | |
|---|---|
| Tickets for the Course - - - - - | $100.00 |
| Demonstrator's Ticket for Anatomy - - - | 5.00 |
| Matriculation Fee, but once - - - - | 5.00 |
| Diploma Fee - - - - - - - | 30.00 |

### TERMS OF GRADUATION.

Two courses (including one of the dissections), with a satisfactory dental pupilage. Eight years reputable practice, or a satisfactory examination upon the branches of the Junior Course in this Institution, will be received as equal to one course.

An acceptable Thesis upon some subject in Dental Science.

Must possess a good moral character, and be twenty-one years old.

For further information, address J. TAFT, Dean,
117 W. Fourth St., Cincinnati, O.

### PENNSYLVANIA COLLEGE OF DENTAL SURGERY.

THE EIGHTEENTH ANNUAL SESSION, 1873-'74.

### FACULTY.

T. L. BUCKINGHAM, D. D. S.,
*Professor of Chemistry.*
E. WILDMAN, M. D., D. D. S.,
*Professor of Mechanical Dentistry and Metallurgy.*
G. T. BAKER, D. D. S
*Professsor of Dental Pathology and Therapeutics.*
JAMES TRUMAN, D. D. S.,
*Professor of Dental Histology and Operative Chemistry.*
JAMES TYSON, M. D.,
*Professor of Physiology and Microscopic Anatomy*
*J. EWING MEARS, M. D.,*
*Professor of Anatomy and Surgery.*

E. R. PETTIT, D. D. S.,
*Demonstrator of Operative Dentistry.*
A, B. ABELL, JR., D. D. S.,
*Demonstrator of Mechanical Dentistry.*
J. L. SAXTON, D. D S.,
*Assistant Demonstrator of Operative Dentistry.*
A. H. HENDERSON, D. D. S.,
*Assistant Demonstrator of Mechanical Dentistry.*

The College will be open for instruction during the entire year, except the last week in August and the first week in September. In the Dispensary and Laboratory, ample opportunities will be afforded the student for the prosecution of the practical part of the profession, under the guidance and supervision of Demonstrators of known integrity and capacity. A Clinical Lecture will be given and operations performed by one of the professors every Saturday afternoon during the sessions.

### SPRING AND FALL SESSIONS.

The Spring Course of Lectures will commence on the first Monday in April, and continue uutil the last of June.

The Fall Course will commence on the second Monday in September, and continue until the last of October,

During the Spring and Fall Sessions there will be one Lecture a day, from 5 to 6 o'clock, P. M., and with the exception of Saturday, the Dispensary and Laboratory will be open daily for six hours.

The fee for the tickets will be $50.

The attendance of the entire Spring and Fall Courses will be deemed equivalent to the term of pupilage under a private preceptor during the recess of the regular sessions.

### THE REGULAR SESSION.

Will commence on the first Monday of November, and continue until the first of March ensuing. The course is so arranged that eighteen lectures will be delivered each week on the various branches taught in the College

FEES.

| | |
|---|---|
| Matriculation, (paid but once,) - - - - - - - - - - - - - | $5 00 |
| For the Course, (Demonstrator's Ticket included,) - - - - - - | 100 00 |
| Diploma, - - - - - - - - - - - - - - - - - - | 30 00 |

For further information, address,

**E. WILDMAN Dean,**

**No. 1205 Arch Street, Philadelphia.**

Board can be obtained at from $4.00 to $8.00 per week.

All the Instruments and Tools required can be procured for from $15.00 to $20.00.

### OHIO COLLEGE
OF
### DENTAL SURGERY,

The Twenty-eighth Annual Session of the Ohio College of Dental Surgery will commence on Monday, the 15th of October next, and continue until March following.

### FACULTY.

JAMES TAYLOR, M. D., D. D S,
Emeritus Professor of Institutes of Dental Science.

C. KEARNS, M. D.,
Professor of Anatomy.

J. S. CASSIDY, D. D. S.,
Professor of Chemistry and Therapeutics.

EDWARD RIVES, M. D.,
Professor of Physiology and Histology.

E. BRUNNING, M. D.,
Professor of Pathology and Therapeutics.

J. TAFT, D. D. S.,
Professor of Operative Dentistry and Dental Hygiene.

FRANK HUNTER, D. D. S.,
Professor of Mechanical Dentistry.

JAMES I. TAYLOR, Jr., D. D. S.,
Professor of Clinical Dentistry.

### TEXT BOOKS.

Gray's and Wilson's Anatomy, Dalton's or Carpenter's Physiology; William's Pathology, Fowne's, Turner's, Graham's, or Brandt and Taylor's Chemistry; Taft's Operative Dentistry; Richardson's Mechanical Dentistry; Harris' Principles and Practice of Dental Science; Tomes' Dental Surgery; Watt's Chemical Essays; Harris' Dental Dictionary.

### TERMS OF ADMISSION.

Tickets must be procured at the beginning of the session.

| | |
|---|---|
| Tickets for the Course - - - - - | $100.00 |
| Demonstrator's Ticket for Anatomy - - - | 5.00 |
| Matriculation Fee, but once - - - - | 5.00 |
| Diploma Fee - - - - - - - | 30.00 |

### TERMS OF GRADUATION.

Two courses (including one of the dissections), with a satisfactory dental pupilage. Eight years reputable practice, or a satisfactory examination upon the branches of the Junior Course in this Institution, will be received as equal to one course.

An acceptable Thesis upon some subject in Dental Science.

Must possess a good moral character, and be twenty-one years old.

For further information, address J. TAFT, Dean,
117 W. Fourth St., Cincinnati, O.

### PENNSYLVANIA COLLEGE OF DENTAL SURGERY.

THE EIGHTEENTH ANNUAL SESSION, 1873-'74.

### FACULTY.

T. L. BUCKINGHAM, D. D. S.,
*Professor of Chemistry.*
E. WILDMAN, M. D., D. D. S.,
*Professor of Mechanical Dentistry and Metallurgy.*
G. T. BAKER, D. D. S
*Professsor of Dental Pathology and Therapeutics.*
JAMES TRUMAN, D. D. S.,
*Professor of Dental Histology and Operative Chemistry.*
JAMES TYSON, M. D.,
*Professor of Physiology and Microscopic Anatomy*
*J. EWING MEARS, M. D.,*
*Professor of Anatomy and Surgery.*
E. R. PETTIT, D. D. S.,
*Demonstrator of Operative Dentistry.*
A, B. ABELL, JR., D. D. S.,
*Demonstrator of Mechanical Dentistry.*
J. L. SAXTON, D. D S.,
*Assistant Demonstrator of Operative Dentistry.*
A. H. HENDERSON, D. D. S.,
*Assistant Demonstrator of Mechanical Dentistry.*

The College will be open for instruction during the entire year, except the last week in August and the first week in September. In the Dispensary and Laboratory, ample opportunities will be afforded the student for the prosecution of the practical part of the profession, under the guidance and supervision of Demonstrators of known integrity and capacity. A Clinical Lecture will be given and operations performed by one of the professors every Saturday afternoon during the sessions.

### SPRING AND FALL SESSIONS.

The Spring Course of Lectures will commence on the first Monday in April, and continue uutil the last of June.

The Fall Course will commence on the second Monday in September, and continue until the last of October,

During the Spring and Fall Sessions there will be one Lecture a day, from 5 to 6 o'clock, P. M., and with the exception of Saturday, the Dispensary and Laboratory will be open daily for six hours.

The fee for the tickets will be $50.

The attendance of the entire Spring and Fall Courses will be deemed equivalent to the term of pupilage under a private preceptor during the recess of the regular sessions.

### THE REGULAR SESSION.

Will commence on the first Monday of November, and continue until the first of March ensuing. The course is so arranged that eighteen lectures will be delivered each week on the various branches taught in the College

FEES.

| | |
|---|---|
| Matriculation, (paid but once,) | $5 00 |
| For the Course, (Demonstrator's Ticket included,) | 100 00 |
| Diploma, | 30 00 |

For further information, address,

**E. WILDMAN Dean,**

**No. 1205 Arch Street, Philadelphia.**

Board can be obtained at from $4.00 to $8.00 per week.

All the Instruments and Tools required can be procured for from $15.00 to $20.00.

### PENNSYLVANIA COLLEGE OF DENTAL SURGERY.

THE EIGHTEENTH ANNUAL SESSION, 1873-'74.

### FACULTY.

T. L. BUCKINGHAM, D. D. S.,
*Professor of Chemistry.*
E. WILDMAN, M. D., D. D. S.,
*Professor of Mechanical Dentistry and Metallurgy.*
G. T. BAKER, D. D. S
*Professsor of Dental Pathology and Therapeutics.*
JAMES TRUMAN, D. D. S.,
*Professor of Dental Histology and Operative Chemistry.*
JAMES TYSON, M. D.,
*Professor of Physiology and Microscopic Anatomy*
*J. EWING MEARS, M. D.,*
*Professor of Anatomy and Surgery.*
E. R. PETTIT, D. D. S.,
*Demonstrator of Operative Dentistry.*
A, B. ABELL, JR., D. D. S.,
*Demonstrator of Mechanical Dentistry.*
J. L. SAXTON, D. D S.,
*Assistant Demonstrator of Operative Dentistry.*
A. H. HENDERSON, D. D. S.,
*Assistant Demonstrator of Mechanical Dentistry.*

The College will be open for instruction during the entire year, except the last week in August and the first week in September. In the Dispensary and Laboratory, ample opportunities will be afforded the student for the prosecution of the practical part of the profession, under the guidance and supervision of Demonstrators of known integrity and capacity. A Clinical Lecture will be given and operations performed by one of the professors every Saturday afternoon during the sessions.

### SPRING AND FALL SESSIONS.

The Spring Course of Lectures will commence on the first Monday in April, and continue uutil the last of June.

The Fall Course will commence on the second Monday in September, and continue until the last of October,

During the Spring and Fall Sessions there will be one Lecture a day, from 5 to 6 o'clock, P. M., and with the exception of Saturday, the Dispensary and Laboratory will be open daily for six hours.

The fee for the tickets will be $50.

The attendance of the entire Spring and Fall Courses will be deemed equivalent to the term of pupilage under a private preceptor during the recess of the regular sessions.

### THE REGULAR SESSION.

Will commence on the first Monday of November, and continue until the first of March ensuing. The course is so arranged that eighteen lectures will be delivered each week on the various branches taught in the College

FEES.

| | |
|---|---|
| Matriculation, (paid but once,) | $5 00 |
| For the Course, (Demonstrator's Ticket included,) | 100 00 |
| Diploma, | 30 00 |

For further information, address,

**E. WILDMAN Dean,**

**No. 1205 Arch Street, Philadelphia.**

Board can be obtained at from $4.00 to $8.00 per week.

All the Instruments and Tools required can be procured for from $15.00 to $20.00.

### OHIO COLLEGE
OF
### DENTAL SURGERY,

The Twenty-eighth Annual Session of the Ohio College of Dental Surgery will commence on Monday, the 15th of October next, and continue until March following.

### FACULTY.

JAMES TAYLOR, M. D., D. D S,
Emeritus Professor of Institutes of Dental Science.

C. KEARNS, M. D.,
Professor of Anatomy.

J. S. CASSIDY, D. D. S.,
Professor of Chemistry and Therapeutics.

EDWARD RIVES, M. D.,
Professor of Physiology and Histology.

E. BRUNNING, M. D.,
Professor of Pathology and Therapeutics.

J. TAFT, D. D. S.,
Professor of Operative Dentistry and Dental Hygiene.

FRANK HUNTER, D. D. S.,
Professor of Mechanical Dentistry.

JAMES I. TAYLOR, Jr., D. D. S.,
Professor of Clinical Dentistry.

### TEXT BOOKS.

Gray's and Wilson's Anatomy, Dalton's or Carpenter's Physiology; William's Pathology, Fowne's, Turner's, Graham's, or Brandt and Taylor's Chemistry; Taft's Operative Dentistry; Richardson's Mechanical Dentistry; Harris' Principles and Practice of Dental Science; Tomes' Dental Surgery; Watt's Chemical Essays; Harris' Dental Dictionary.

### TERMS OF ADMISSION.

Tickets must be procured at the beginning of the session.

| | |
|---|---|
| Tickets for the Course - - - - - | $100.00 |
| Demonstrator's Ticket for Anatomy - - - | 5.00 |
| Matriculation Fee, but once - - - - | 5.00 |
| Diploma Fee - - - - - - - | 30.00 |

### TERMS OF GRADUATION.

Two courses (including one of the dissections), with a satisfactory dental pupilage. Eight years reputable practice, or a satisfactory examination upon the branches of the Junior Course in this Institution will be received as equal to one course.

An acceptable Thesis upon some subject in Dental Science.

Must possess a good moral character, and be twenty-one years old.

For further information, address J. TAFT, Dean,
117 W. Fourth St., Cincinnati, O

